# Predictors for selective flexure mobilization during robotic anterior resection for rectal cancer: a prospective cohort analysis

**DOI:** 10.1007/s00464-023-10008-x

**Published:** 2023-04-03

**Authors:** Jeremy Meyer, George van der Schelling, Jan Wijsman, Frédéric Ris, Rogier Crolla

**Affiliations:** 1 Division of Digestive Surgery, University Hospitals of Geneva, Rue Gabrielle-Perret-Gentil 4, 1211 Geneva 14, Switzerland; 2 Medical School, University of Geneva, Rue Michel-Servet 1, 1206 Geneva, Switzerland; 3 Amphia Ziekenhuis, Molengracht 21, 4818CK Breda, Netherlands

**Keywords:** Robotics, Da Vinci, TME, Low anterior resection, LAR, Total mesorectum excision

## Abstract

**Introduction:**

Splenic flexure mobilization (SFM) may be indicated during anterior resection to provide a tension-free anastomosis. However, to date, no score allows identifying patients who may benefit from SFM.

**Methods:**

Patients who underwent robotic anterior resection for rectal cancer were identified from a prospective register. Demographic and cancer-related variables were extracted, and predictors of SFM were identified using regression models. Thereafter, 20 patients with SFM and 20 patients without SFM were randomly selected and their pre-operative CTscan were reviewed. The radiological index was defined as 1/(sigmoid length/pelvis depth). The optimal cut-off value for predicting SFM was identified using ROC curve analysis.

**Results:**

Five hundred and twenty-four patients were included. SFM was performed in 121 patients (27.8%) and increased operative time by 21.8 min (95% CI: 11.3 to 32.4, p < 0.001). The incidence of postoperative complications did not differ between patient with or without SFM. Realization of an anastomosis was the main predictor for SFM (OR: 42.4, 95% CI: 5.8 to 308.5, p < 0.001). In patients with colorectal anastomosis, both sigmoid length (15 ± 5.1 cm versus 24.2 ± 80.9 cm, p < 0.001) and radiological index (1 ± 0.3 versus 0.6 ± 0.2, p < 0.001) differed between patients who had SFM and patients who did not. ROC curve analysis of the radiological index indicated an optimal cut-off value of 0.8 (sensitivity: 75%, specificity: 90%).

**Conclusion:**

SFM was performed in 27.8% of patients who underwent robotic anterior resection, and increased operative time by 21.8 min. For optimal surgical planning, patients requiring SFM can be identified based on pre-operative CT using the index 1/(sigmoid length/pelvis depth) with a cut-off value set at 0.8.

**Supplementary Information:**

The online version contains supplementary material available at 10.1007/s00464-023-10008-x.

Splenic flexure mobilization (SFM) separates the mesocolon from its posterior attachments to the pancreas and the Gerota’s fascia following the embryological planes, and opens the *bursa omentalis* [1, 2]. SFM allows accessing the retroperitoneal structures and, in colorectal surgery, resecting splenic flexure cancer or medializing the colon for providing additional length of colonic conduit. This is notably of importance when a tension-free colorectal anastomosis has to be performed, such as in case of low anterior resection, but also in case of colostomy formation during Hartmann or abdomino-perineal excision in obese patients with important subcutaneous fat. Cadaveric study revealed that SFM with ligation of the IMV at the inferior border of the pancreas allowed providing additional 18 ± 6.8 cm of colonic conduit from the colo-sigmoid junction to the pubic symphysis, whereas only 5 ± 5.5 cm were obtained after high ligation of the inferior mesenteric artery without SFM [3].

However, SFM increases operating time [4–8] by up to 10% [9] and exposes the patients to risk of iatrogenic injuries [4, 10]. Of note, the incidences of surgical site infection [11], pancreatic tail injury and splenic injury [10] are increased in patients undergoing SFM. Therefore, depending on local guidelines, SFM may be reserved to selected patients [4, 6, 7, 12].

Considering that SFM is seen as a technically challenging step when performing anterior resection, most surgeons feel more comfortable starting the procedure with SFM, rather than noting an insufficient colonic conduit length at the end of total mesorectum excision and having to perform SFM in a second step. Moreover, as SFM increases the operative time, knowing if SFM is required or not beforehand may allow improving surgical planning and resources allocation.

Therefore, based on the experience of a center performing selective SFM during robotic anterior resection for colorectal cancer, we aimed at identifying patients requiring SFM based on pre-operative variables.

## Methods

### Inclusion process

The study was performed using a prospective cohort of robotic colorectal resections performed in a single centre from March 2012 to September 2022. Patients who underwent robotic proctectomy with partial mesorectum excision or total mesorectum excision for rectal cancer, including high anterior resection, low anterior resection and abdomino-perineal resection, were considered for inclusion. To this end, the database was reviewed, and patients who had another diagnosis than colorectal cancer (such as diverticular disease, inflammatory bowel disease or anal cancer), those who underwent another colorectal resection than including partial mesorectum excision or total mesorectum excision (such as right hemicolectomy, left hemicolectomy, segmental transverse colectomy or sigmoid colectomy) and those who received a combined procedure (such as rectal surgery associated with small bowel resection, or pelvic exenteration) were excluded. Patients with colorectal cancer localized at more than 15 cm from the anal verge based on preoperative staging MRI, or those with missing data from preoperative staging MRI, were also excluded.

### Surgical procedure

Anterior resection with partial or total mesorectum excision, or abdomino-perineal excision, were performed as previously reported [1]. SFM was performed, if required, and using a totally robotic approach. If SFM was performed, the inferior mesenteric vein was ligated at the inferior border of the pancreas. The medial-to-lateral or lateral-to-medial approaches were used [2], according to surgeons’ preferences. Arterial control and extent of lymphadenectomy (D2 or D3) were left to surgeons’ preferences. If possible, the left colic artery was preserved, and the superior rectal artery was divided at its origin. A circular stapled anastomosis was performed, if indicated, and protected or not by a loop ileostomy. Surgical procedures were performed using the Da Vinci Si or Da Vinci Xi surgical robots.

### Demographic variables

Age, sex, ASA score at time of surgery, body mass index (BMI) at time of surgery, tumor height based on initial preoperative staging MRI, neo-adjuvant radiotherapy, restoration of the bowel continuity (anastomosis) and final pathologic TNM stage were extracted from the database. Patients were subdivided into non-obese (BMI < 30 kg/m^2^) and obese (BMI ≥ 30 kg/m^2^) categories. Obesity was further subdivided into class I (30.0–34.9 kg/m^2^), class II (35.0–39.9 kg/m^2^) and class III (> 40 kg/m^2^). Rectal cancer was subdivided as follows, based on staging MRI findings: high rectum: 10-15 cm from the anal verge, mid rectum: 5-10 cm from the anal verge and low rectum: < 5 cm from the anal verge.

### Radiological variables

**Fig. 1:**
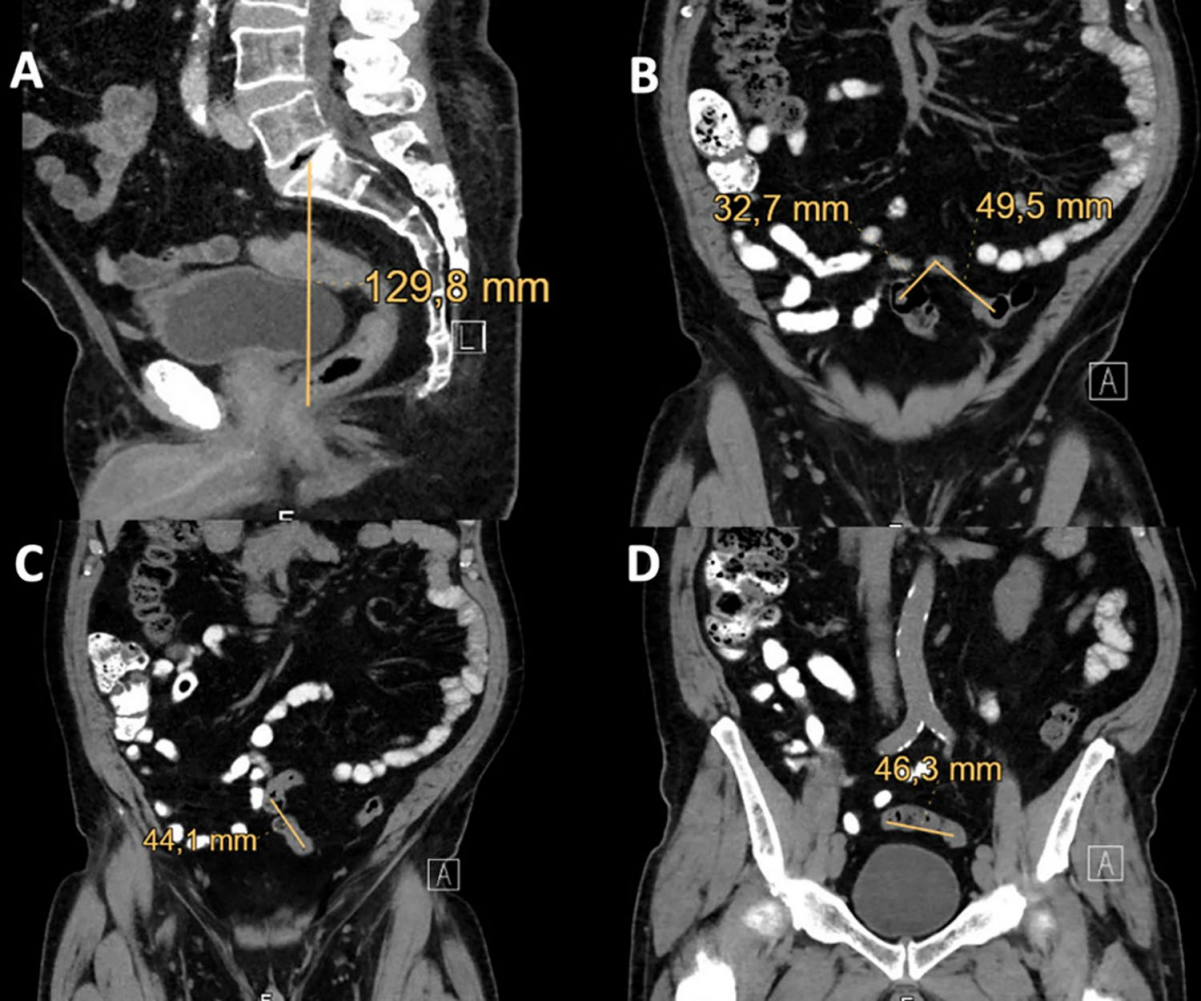
Methods for measuring the sigmoid length. **A** Sagittal plane: The pelvis depth was estimated by measuring the distance from the middle of the top of the first sacral vertebra (S1) to the passage of the rectum through the pelvic floor. **B**–**D** Coronal plane: The sigmoid length was measured, in several planes, from the crossing with the psoas muscle to the recto-sigmoid junction

A random sample of 20 patients with SFM and 20 patients without SFM was generated from patients with colorectal anastomosis. Patients’ identifiers were extracted and staging CTscan were reviewed by an investigator blinded for the operative note (and the occurrence of SFM or not). The length of the sigmoid was arbitrarily estimated in the coronal plane, starting at the level of the large bowel crossing the psoas muscle and ending at the top of the rectum, defined as the sigmoid take-off. The depth of the pelvis was estimated in the sagittal plane by measuring the distance from the middle of the top of the first sacral vertebra (S1) to the passage of the rectum through the pelvic floor (Fig. 1). The index was calculated as follows: 1/(length of the sigmoid/depth of the pelvis).

### Statistical analysis

Differences in terms of demographic, cancer and radiological variables between patients with and without SFM during anterior resection with partial or total mesorectum excision were compared using the two-sided Student’s test, the Pearson’s chi-squared test or the Fisher’s exact test, as appropriate. Continuous variables were transformed into categorical variables if required. Variables were expressed as proportions for categorical variables and means for continuous ones; 95% confidence intervals (95% CI) and standard deviations (SD) were reported. Calculation of proportions accounted for missing data.

Pre-operative predictors of SFM were identified by performing univariate logistic regression, considering as the dependent variable the occurrence (or not) of SFM, and as dependent variables the demographic variables, the cancer variables, the estimated length of sigmoid and the radiological index. Subgroups analyses were notably performed according to tumour height and type of surgical procedure.

A receiver operating characteristics (ROC) curve was drawn to define the optimal cut-off point of the radiological index allowing to identify patients requiring SFM. The optimal cut-off point was determined using the Liu method using a STATA add-on.

All statistical analyses were performed using STATA (version 17, StataCorp LP, College Station, USA). The null hypothesis was rejected at *p* < 0.05.

### Ethics

Institutional review board approval was granted.

## Results

### Patients’ selection

**Fig. 2:**
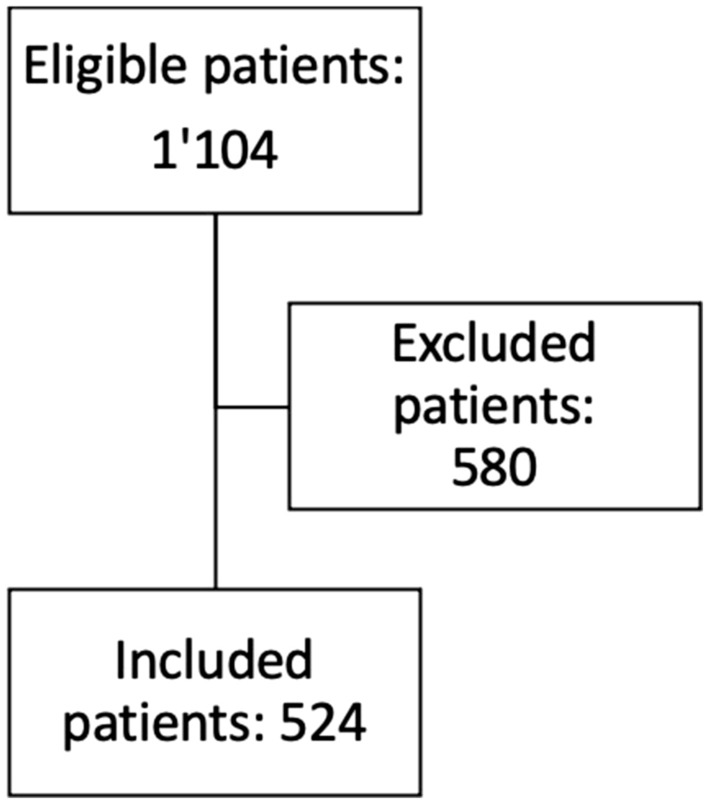
Inclusion flowchart. Patients were included if they underwent robotic anterior resection or abdomino-perineal excision for rectal cancer

One-thousand one-hundred and four patients underwent robotic colorectal surgery over the 10.5-year study period (03.2012–09.2022). Five-hundred and eighty patients were excluded. Based on database analysis, reasons for exclusion were the following: another surgical procedure than anterior resection (137 patients), another diagnosis than colorectal cancer (102 patients: 63 patients with diverticular disease, 8 patients with inflammatory bowel disease, 4 patients with anal cancer and 27 patients with other diagnoses), anterior resection associated with another procedure (pelvic exenteration) and/or with resection of another bowel segment (102 patients). Based on MRI review, 239 patients were further excluded for having a tumor localized more than 15 cm from the anal verge or because of missing MRI data. Ultimately, 524 patients were included (Fig. 2).

### Description of the cohort

The mean age was 66.9 ± 11 years. Three-hundred and forty-five patients (65.8%) were males. The mean BMI was 26.3 ± 4.6 kg/m^2^, and 92 patients (17.6%) were obese. Based on pre-operative MRI, 64 patients (12.2%) had high rectal cancer, 216 (41.2%) had mid rectal cancer and 244 (46.6%) had low rectal cancer.

Four-hundred and eighty-three (92.2%) patients were operated using the Da Vinci Si, and 41 (7.8%) using the Da Vinci Xi (Intuitive Surgicals, Sunnyvale, USA). Three-hundred and fifty-four (68.5%) patients had anterior resection with partial or total mesorectum excision, and 170 (32.4%) had abdomino-perineal excision. Fluorescence angiography was performed in 298 patients (56.9%). An anastomosis was done in 355 patients (67.9%). Among these patients, the anastomosis was side-to-end in 199 patients (51.6%), end-to-end in 66 patients (17.1%) and side-to-side in 1 patient (0.3%). One-hundred and seventy-three patients (33%) had a protection loop ileostomy. The robotic procedure had to be converted to open surgery in 4 patients (0.8%). The mean duration of the surgical procedure was 221.2 ± 50.7 min (213.7 ± 50 min for anterior resection with partial or total mesorectum excision and 236.8 ± 48.7 min for abdomino-perineal excision). The mean length of stay was 8.2 ± 8 days. Demographic, surgical and cancer-related variables are summarized in Table 1.

**Table 1:** Characteristics of the study population

	Total (*n* = 524)	SFM (*n* = 121, 27.8%)	No SFM (*n* = 315, 72.3%)	*p*-value
Males, *n*(%)	345 (65.8%)	86 (71.1%)	197 (62.5%)	0.095
Age (years), mean ± SD	66.9 ± 11	65 ± 10.6	67.1 ± 11.3	0.075
ASA, *n* (%)				
I	79 (15.1%)	23 (19%)	45 (14.3%)	0.189
II	300 (57.4%)	73 (60.3%)	176 (56.1%)	
III	140 (26.8%)	25 (20.7%)	90 (28.7%)	
IV	4 (0.8%)	–	3 (1%)	
BMI, median ± SD	26.2 ± 4.3	26.1 ± 3.9	26.3 ± 4.4	0.669
Obesity, *n* (%)	92 (17.6%)	20 (16.5%)	57 (18.1%)	0.701
Class I	74 (14.2%)	18 (14.9%)	44 (14.1%)	0.547
Class II	12 (2.3%)	1 (0.8%)	9 (2.9%)	
Class III	2 (0.4%)	1 (0.8%)	1 (0.3%)	
Class IV	1 (0.2%)	–	–	
Neo-adjuvant radiotherapy, *n* (%)	295 (56.3%)	73 (60.3%)	162 (51.4%)	0.095
Tumor height, *n* (%)				
High	64 (12.2%)	19 (15.7%)	45 (14.3%)	0.678
Mid	216 (41.2%)	61 (50.4%)	149 (47.3%)	
Low	244 (46.6%)	41 (33.9%)	121 (38.4%)	
Anastomosis, *n* (%)	355 (67.9%)	120 (99.2%)	232 (73.9%)	< 0.001
TNM stage				
Stage I, *n* (%)	171 (33%)	37 (30.8%)	100 (31.8%)	0.988
Stage II, *n* (%)	142 (27.4%)	34 (28.3%)	93 (29.6%)	
Stage III, *n* (%)	153 (29.5%)	37 (30.8%)	90 (28.7%)	
Stage IV, *n* (%)	34 (6.6%)	8 (6.7%)	19 (6.1%)	

Differences between patients with or without SFM were estimated using the two-sided Student’s test or the Pearson’s chi-squared test, as appropriate. Continuous variables were transformed into categorical variables if required. Variables were expressed as proportions for categorical variables and means for continuous ones; 95% confidence intervals (95% CI) and standard deviations (SD) were reported. Calculation of proportions accounted for missing data. Numbers do not necessarily add up for some variables if there were missing data. *SFM* splenic flexure mobilization

### Splenic flexure mobilization

SFM was performed in 121 patients (27.8%). Age, sex, ASA score, BMI, neo-adjuvant radiotherapy, tumor height based on preoperative MRI and pTNM stage did not differ between patients who benefited from SFM and those who did not, as shown by Table 1. However, patients in which an anastomosis was performed were more likely to undergo SFM than patients in whom an anastomosis was not performed (34.1% versus 1.2%, *p* < 0.001, respectively).

The operative time was longer in patients who had SFM than in patients who did not have SFM (233.5 ± 45.9 min versus 211.7 ± 51.1, *p* < 0.001, respectively). This difference was maintained in patients in whom an anastomosis was performed (232.9 ± 45.5 min versus 203.5 ± 49.2 min, *p* < 0.001, respectively), as reported in Table 2. Using linear regression with operative time set as the dependent variable, performing SFM added 21.8 min (95% CI: 11.3 to 32.4, *p* < 0.001) to the surgical procedure, and 29.4 min (95% CI: 18.7 to 40.1, *p* < 0.001) in patients with realization of a colorectal anastomosis.

**Table 2:** Operative time

	Total (*n* = 524)	SFM (*n* = 121, 27.8%)	No SFM (*n* = 315, 72.3%)	*p*-value
Anterior resection with PME/TME	221.2 ± 50.7	233.5 ± 45.9	211.7 ± 51.1	< 0.001
High rectal cancer	184.3 ± 40.9	214.5 ± 35.7	171.3 ± 36	< 0.001
Mid rectal cancer	215.9 ± 50.4	232.1 ± 48.8	209.4 ± 49.7	0.003
Low rectal cancer	233.3 ± 47.7	244.4 ± 43.5	229.5 ± 48.6	0.084
With anastomosis	213.5 ± 49.9	232.9 ± 45.5	203.5 ± 49.2	< 0.001

Differences between patients with or without SFM were estimated using the two-sided Student’s test. Variables were expressed as means; standard deviations (SD) were reported. *SFM* splenic flexure mobilization

### Post-operative complications

Overall, 112 patients (21.3%) developed at least one post-operative complication after robotic mesorectum excision. Complications were graded as follows: 7.3% grade I, 17.8% grade II, 19.9% grade III and 1.6% grade IV according to the Clavien-Dindo classification. Thirty-day mortality was of 2.4% (9 patients). The incidence of anastomotic leak was of 47 patients (13.2%, among patients who had an anastomosis). The 30-day incidence of post-operative complications, 30-day incidence of anastomotic leak, 30-day incidence mortality did not differ between patients with SFM and in those without SFM (Table S1).

### Predictors of splenic flexure mobilization

The incidence of SFM was 27.8%. Considering that performing SFM requires additional time, reserving it to a subpopulation of selected patients may allow saving hospital resources. Therefore, we aimed at identifying predictors of SFM during robotic anterior resection. However, neither age, sex, ASA class, BMI, obesity category, neo-adjuvant radiotherapy, tumor localization from the anal verge based on preoperative MRI and pTNM score were identified as predictors for SFM based on logistic regression, as reported in Table 3. Nonetheless, comparative analysis showed that significantly more patients with anastomosis (34.1%) had to undergo SFM than patients without anastomosis (1.2%). This was confirmed by logistic regression, as realizing an anastomosis predicted the necessity for SFM (OR: 42.4, 95% CI: 5.8 to 308.5, *p* < 0.001). Subgroup analysis was performed including only patients with colorectal anastomosis (therefore excluding those with end stoma and/or APE), as reported in Table 3. In these patients, previous radiotherapy allowed predicting the necessity for SFM (OR: 1.66, 95% CI: 1.06 to 2.60, *p* = 0.026).

**Table 3:** Identification of predictors of splenic flexure mobilization

		Total (*n* = 524)		Patients with anastomosis(*n* = 355, 67.9%)	
		OR (95% CI)	*p*-value	OR (95% CI)	*p*-value
Age	Continuous (years)	0.98 (0.97–1)	0.077	1 (0.98–1.02)	0.787
Age	< 50 years	Reference	–	Reference	–
	50–75 years	0.54 (0.25–1.18)	0.121	0.60 (0.27–1.35)	0.217
	> 75 years	0.46 (0.19–1.09)	0.078	0.69 (0.28–1.70)	0.421
Males	Yes	1.47 (0.93–2.32)	0.096	1.54 (0.96–2.47)	0.075
ASA	I	References	–	References	–
	II	0.81 (0.46–1.44)	0.474	0.92 (0.51–1.66)	0.789
	III	0.54 (0.28–1.06)	0.074	0.89 (0.44–1.80)	0.748
	IV	–	–	–	–
BMI	Continuous (kg/m2)	0.99 (0.94–1.04)	0.668	1 (0.95–1.05)	0.948
Obesity	No obesity	References	–	References	–
	Class I	1.05 (0.58–1.89)	0.885	1.22 (0.63–2.33)	0.558
	Class II	0.28 (0.04–2.27)	0.235	0.28 (0.03–2.27)	0.231
	Class III	2.55 (0.16–41.23)	0.509	1.93 (0.12–31.19)	0.643
	Class IV	–	–	–	–
Previous radiotherapy	Yes	1.44 (0.94–2.20)	0.096	1.66 (1.06–2.60)	0.026
Tumor height	High	References	–	References	–
	Mid	0.97 (0.53–1.79)	0.921	1.05 (0.57–1.95)	0.866
	Low	0.80 (0.42–1.53)	0.502	1.89 (0.96–3.73)	0.065

Potential predictors of SFM were looked at using univariate logistic regression. Continuous variables were transformed into categorical variables if required. Odds ratios (OR) and 95% confidence intervals (95% CI) were reported

### Radiological predictors of splenic flexure mobilization

Considering that demographic and tumor-related variables did not allow identifying the subpopulation of rectal cancer patients who may require SFM, and that performing this additional step in every patient with anastomosis is not necessary (72.3% of patients did not need SFM), we aimed at identifying radiological predictors of SFM. Based on our personal experience, we hypothesized that a pre-existing long sigmoid provided enough length for the colonic conduit for performing a tension-free anastomosis without compromising oncological safety in patients with rectal cancer, notably if the left colic artery was preserved and if perfusion was checked using ICG fluorescence angiography. Therefore, from patients who had an anastomosis (therefore excluding patients with end stoma and/or abdomino-perineal excision), we randomly selected 20 patients with SFM and 20 patients without SFM and blindly reviewed their preoperative CTscan. This subgroup of patients comprised 70% males (28 patients), who had a mean age of 65.5 ± 8.9 years and a mean BMI of 26.1 ± 4.1 kg/m2. Sixteen (40%) of them had received neo-adjuvant radiotherapy. Based on staging pelvic MRI, their tumors were localized as follow: high rectum in 5 patients (12.5%), mid rectum in 22 patients (55%) and low rectum in 13 patients (32.5%). Thirteen patients (32.5%) had stage I rectal cancer, 14 (35%) had stage II rectal cancer, 12 (30%) had stage III rectal cancer and 1 (2.5%) had stage IV rectal cancer.

**Fig. 3:**
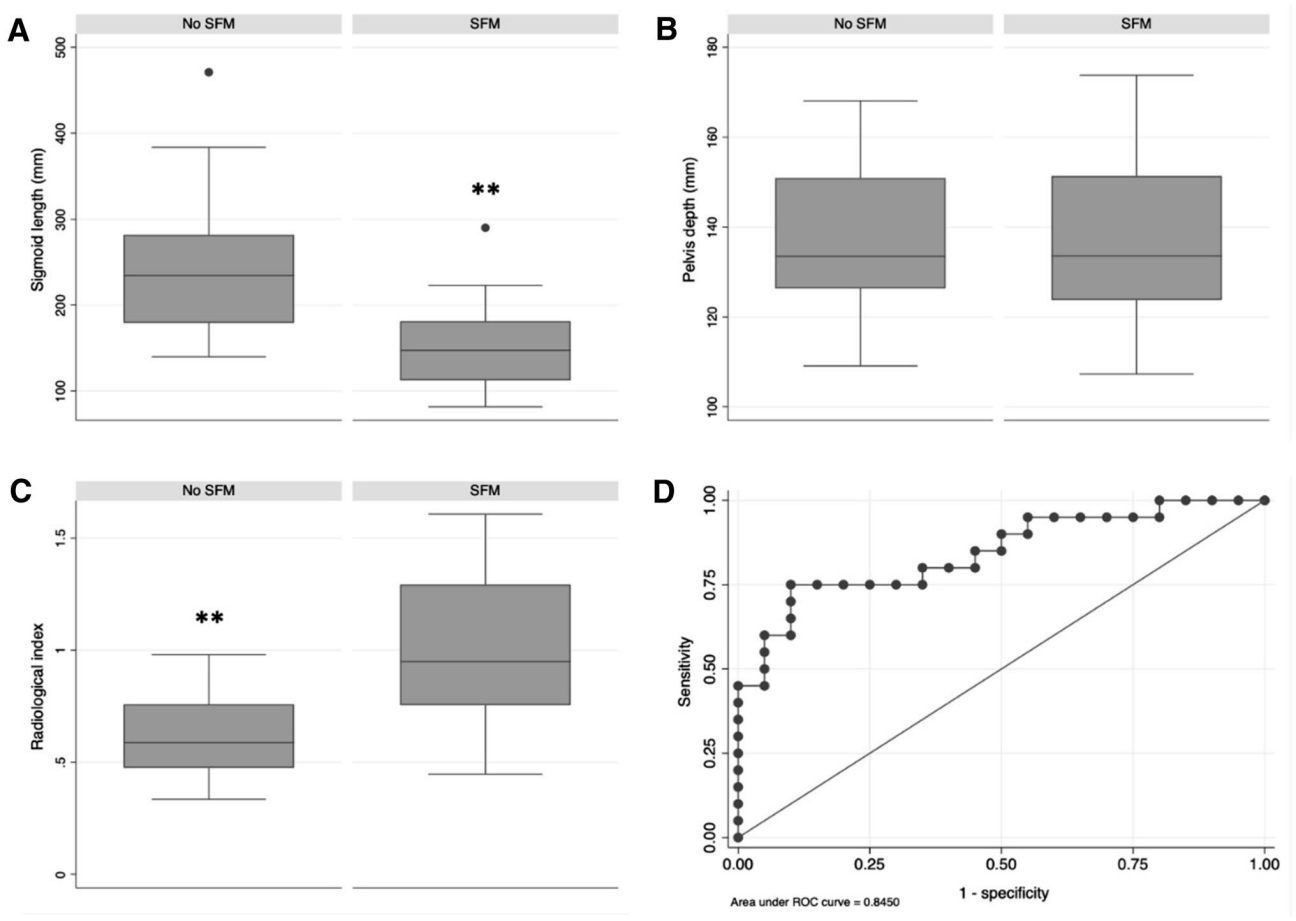
Analysis of radiological variables for predicting the necessity of SFM. Data are represented as boxplots: the horizontal line represents the median, the square indicates the SD, whiskers represent 95% confidence intervals. P-values were obtained using the Student’s *t*-test. **A** Length of the sigmoid, **B** Pelvis depth, **C** Radiological index, defined as: 1/(length of the sigmoid/depth of the pelvis), **D** Receiver operating characteristics (ROC) curve showing the ability of the radiological index for predicting SFM. *SFM* splenic flexure mobilization

Among this subset of patients, patients who had SFM had shorter sigmoid length (15 ± 5.1 cm versus 24.2 ± 8.1 cm, *p* < 0.001) than patients who did not required SFM. The pelvis depth was the same between these two groups of patients. The radiological index was higher in patients with SFM (1 ± 0.3 versus 0.6 ± 0.2, *p* < 0.001) than in patients without SFM (Table 4, Fig. 3). According to logistic regression, both the estimation of the sigmoid length (OR: 0.97, 95% CI: 0.96 to 0.99, *p* = 0.002) and the radiological index (OR: 364.9, 95%CI: 8.2 to 16244.4, *p* = 0.002) allowed predicting the need or not for SFM. Sensitivity analysis revealed that the radiological index was significantly different between patients with or without SFM in the subgroup of patients with low rectal cancer based on staging MRI (Table 4). The radiological index allowed predicting the necessity for SFM with an area under the curve of 84.5% (95% CI: 72.2 to 96.8%). For a cut-off value optimally set at 0.8 according to the Liu’s methods, the sensitivity, specificity, positive predictive value and negative predictive value for predicting SFM were of, respectively, 75, 90, 88.2 and 78.3% (Fig. 3).

**Table 4:** Radiological measurements

		Total (*n* = 40)	SFM (*n* = 20)	No SFM (*n* = 20)	*p*-value
Sigmoid length (mm), mean ± SD	Anterior resection with PME/TME	195.9 ± 81.3	150.1 ± 51.2	241.7 ± 80.9	< 0.001
Pelvis depth (mm), mean ± SD	Anterior resection with PME/TME	136.6 ± 16.8	136.7 ± 18.4	136.6 ± 15.6	0.979
Radiological index, mean ± SD	Anterior resection with PME/TME	0.8 ± 0.3	1 ± 0.3	0.6 ± 0.2	< 0.001
	High rectal cancer	0.8 ± 0.4	1.2 ± 0.6	0.6 ± 0.1	0.214
	Mid rectal cancer	0.8 ± 0.3	0.9 ± 0.3	0.7 ± 0.2	0.050
	Low rectal cancer	0.9 ± 0.4	1.1 ± 0.2	0.5 ± 0.2	< 0.001

The length of the sigmoid length was measured in the coronal plane, starting at the level of crossing the psoas muscle and ending at the top of the rectum. The depth of the pelvis was estimated in the sagittal plane by measuring the distance from the middle of the top of the first sacral vertebra (S1) to the passage of the rectum through the pelvic floor. The radiological index was calculated as follows: 1/(length of the sigmoid/depth of the pelvis). Differences between patients with or without SFM were estimated using the two-sided Student’s test. Variables were expressed as means; standard deviations (SD) were reported. *SFM* splenic flexure mobilization

## Discussion

Performing selective SFM during anterior resection has become standard practice in most centers. However, selection of patients is based on the subjective appreciation of the operating surgeon and, so far, no objective scoring system allows determining what patient may or may not require SFM.

The incidence of SFM reported in the literature varies widely, with some teams reporting performing SFM in only 4% of patients who underwent laparoscopic anterior resection [12], whereas cohorts with bigger sample sizes, such as the analysis of the 2005–2016 NSQIP database reported that SFM was done in 41.6% of patients [13]. In our prospective cohort study including 524 patients who underwent robotic mesorectum excision for rectal cancer, SFM was performed in 121 patients (27.8%). This heterogeneity in the incidence of SFM highlights the absence of strict criteria for identifying patients who require the procedure.

Predicting the necessity of performing SFM is of importance as, in our cohort, SFM added 29.4 min to the surgical procedure if a colorectal anastomosis was performed. Of note, the wide 95% CI (from 18.7 to 40.1 min) may be explained by the heterogeneity in the anatomy of the splenic flexure, which was shown to modulate the duration of this additional procedure step [14]. Analysis of 66,068 patients from the NSQIP database found that adding SFM increased operative time from 184 to 220 min (difference of 36 min) [13], and therefore support our findings.

However, demographic or cancer-related variables did not differ between patients who had SFM and those who did not have SFM. Therefore, selection of patients for SFM solely based on demographic variables, such as BMI, or cancer-related variables, such as tumor height based on staging MRI, would not allow identifying patients requiring SFM. Noteworthy, the incidence of SFM was higher in patients in which a colorectal anastomosis was performed (34.1% versus 1.2%, *p* < 0.001), and realizing an anastomosis increased the probability of requiring SFM by more than 40-fold (OR: 42.4, 95% CI: 5.8 to 308.5, *p* < 0.001). This may be explained by the necessity of having a sufficient length of colonic conduit for performing a tension-free colorectal anastomosis and, by that, avoiding a potential risk of anastomotic leak.

Considering that demographic and tumor-related variables did not allow identifying patients requiring SFM, we performed an analysis of radiological variables in a randomly generated subset of patients. Radiological variables were defined based on the personal experience of the authors. Analysis of the radiological variables showed that patients in whom SFM had to be performed had a shorter estimated sigmoid length (15 cm versus 24.2 cm, *p* < 0.001). In order to take into account the patient’s morphology, we plotted this value as an inverse ratio with the pelvis depth, and defined this value as the radiological index. This radiological index was higher in patients with SFM (1 versus 0.6, *p* < 0.001) than in patients without SFM, and every increment of 0.1 unit of this index increased the “risk” of SFM by 36.5-fold. The ideal threshold of the radiological index was determined to be of 0.8. Using this value as a threshold allows predicting the necessity of performing SFM with a sensitivity of 75% and a specificity of 90%. Therefore, if the radiological index is above 0.8 in a patient scheduled for robotic anterior resection, there is a 88.2% “chance” that this patient will require SFM.

The strengths of this study are the following: (1) its large sample size of robotic mesorectum excisions performed by expert surgeons with a standard practice (which limits heterogeneity in selecting patients for SFM and methods for performing SFM); (2) its prospective data recording, which limits the proportion of missing values; (3) its fully minimally invasive aspect, with a low conversion rate of 0.8% which, again, limits its heterogeneity. The limitations of this study are the following: (1) its limited external validity to expert centers in robotic; (2) radiological measurements constituted an estimation of the sigmoid length for the purpose of this study, and did not perfectly reflect the true length of the sigmoid and were not performed in 3-dimensions; (3) its conclusion does not apply to sigmoid colectomy, where the vascular anatomy of the inferior mesenteric artery and its branches may play a significant role in the decision to perform, or not, SFM; and finally, (4) the pilot analysis (and sample size) of the radiological index requires confirmation by an external prospective cohort.

## Conclusion

Considering that SFM increases operative time by 21.8 min, selecting patients who require this additional procedure step is of logistic and economic importance. Based on our prospective cohort, SFM was required in approximately one quarter of patients who underwent robotic mesorectum excision. These patients could be identified based on pre-operative CT using the radiological index 1/(sigmoid length/pelvis depth) with an optimal cut-off value set at 0.8.

## Supplementary Information

Below is the link to the electronic supplementary material.

Supplementary file1 (DOCX 16 KB)—**Table S1** 30-day incidence of post-operative complications. *Among patients who had a colorectal anastomosis. **Calculation of proportions accounted for missing data**. **Numbers do not necessarily add up for some variables if there were missing data. **SFM: splenic flexure mobilization

## Abbreviations

CI: Confidence interval
OR: Odds ratio
PME: Partial mesorectal excision
SD: Standard deviation
SFM: Splenic flexure mobilization
TME: Total mesorectal excision
