# Supplementary material for: Predictors for selective flexure mobilization during robotic anterior resection for rectal cancer: a prospective cohort analysis
Source: Surg Endosc. 2023 Apr 3;37(7):5388–96. doi: 10.1007/s00464-023-10008-x (PMC10322756; doi:10.1007/s00464-023-10008-x)
Supplement: Supplementary file 1 — Supplementary file1 (DOCX 16 KB)—Table S1 30-day incidence of post-operative complications. *Among patients who had a colorectal anastomosis. Calculation of proportions accounted for missing data. Numbers do not necessarily add up for some variables if there were missing data. SFM: splenic flexure mobilization [file 464_2023_10008_MOESM1_ESM.docx]

|  | Total (n=524) | SFM (n=121, 27.8%) | No SFM (n=315, 72.3%) | p-value |
| --- | --- | --- | --- | --- |
| Post-operative complications | 112 (21.3%) | 23 (19%) | 72 (22.9%) | 0.383 |
| Anastomotic leak | 47 (13.2%)* | 14 (11.7%)* | 33 (14.2%)* | 0.494 |
| Mortality | 9 (1.7%) | 2 (2.5%) | 5 (1.9%) | 0.751 |
